# Chromogenic *in situ* hybridization technique for detecting porcine circovirus 3 in lung and lymphoid tissues

**DOI:** 10.14202/vetworld.2023.1444-1450

**Published:** 2023-07-09

**Authors:** Chew Yee Tan, Kah Chun Lee, Ming-Tang Chiou, Chao-Nan Lin, Peck Toung Ooi

**Affiliations:** 1 Department of Veterinary Clinical Studies, Faculty of Veterinary Medicine, Universiti Putra Malaysia, 43400, Serdang, Selangor, Malaysia; 2 Animal Disease Diagnostic Center, College of Veterinary Medicine, National Pingtung University of Science and Technology, Pingtung, Taiwan; 3 Department of Veterinary Medicine, College of Veterinary Medicine, National Pingtung University of Science and Technology, Pingtung, Taiwan

**Keywords:** chromogenic *in situ* hybridization, *in situ* hybridization, porcine circovirus type 3, porcine circoviruses, pigs

## Abstract

**Background and Aim:**

Porcine circovirus 3 (PCV3) was recently reported in Malaysian commercial pig population in 2020 by conventional polymerase chain reaction (PCR), revealing a molecular prevalence of 17.02% in the sampled domestic pig population. This study aims to describe a chromogenic in situ hybridization (ISH) technique using digoxigenin (DIG)-labeled cloned PCV3 open reading frame 1 (ORF1) fragment DNA to detect and localize the PCV3 antigen in formalin-fixed, paraffin-embedded lung, and lymphoid tissue specimens.

**Materials and Methods:**

Since PCV3 was mainly detected in lung and lymphoid tissues, we obtained tissue specimens from these organs from the previous Malaysian PCV3 study. Digoxigenin-labeled ISH probes were designed to target a 69 bp region of PCV3 ORF1 spanning from the nucleotide positions (282–350).

**Results:**

Light microscopy analysis revealed that chromogenic staining of PCV3 antigens was visualized within the cytoplasm of pneumocytes and lymphocytes, indicating positive ISH results. The results of molecular detection of PCV3 using PCR and ISH showed a high agreement of 90.91%, including for the negative PCV3 status for all samples.

**Conclusion:**

This study reports a chromogenic ISH technique using DIG-labeled probes targeting PCV3 ORF1 to detect PCV3 antigens in lung and lymphoid tissues. Despite the limited availability of PCV3 antibodies, ISH remains relevant for investigating PCV3 replication and pathogenesis and can be used complementarily with PCR for evaluating the localization of antigens in infected tissues.

## Introduction

In the past two decades, porcine circovirus type 2 (PCV2), the key etiological agent in PCV2-associated disease (PCVAD), has caused significant economic losses in the pig industry. While the impact of PCVAD on pig production is still being mitigated, a novel PCV species has been discovered. Porcine circovirus 3 (PCV3) was first reported in 2015 in a US sow herd with increased mortality and decreased conception rate. The sows in the index case presented clinical signs similar to porcine dermatitis and nephropathy syndrome (PDNS), and reproductive failure, which were often associated with PCV2. Metagenomic analysis revealed a novel PCV, designated PCV3 [1]. Another report described PCV3 cases in weaners showing myocarditis with multisystemic inflammation [2]. Although PCV3 infection is associated with multiple clinical signs, the three most consistent clinical presentations are reproductive failure, multisystemic inflammation, and subclinical infection [3]. While many field reports indicated coinfections of PCV3 with other common swine pathogens, the pathogenicity of PCV3 as a single etiological agent was demonstrated through successful reproduction of PDNS-like clinical disease following experimental inoculation of 4- and 8-week-old specific-pathogen-free piglets with infectious PCV3 DNA clone [4]. Recently, another novel species was identified in China, South Korea, Thailand, and Malaysia, sequentially named PCV4 [5–8]. Although the infectious PCV4 clones induced systemic pathological changes in piglets, it may be too early to corroborate the clinical significance of PCV4 in swine husbandry [9].

PCV3 infection continues to be reported in more countries, showing the prevalence of PCV3 coinfection with other ubiquitous porcine pathogens [10]. Numerous polymerase chain reaction (PCR) and quantitative-PCR (qPCR)-based PCV3 diagnostic assays have been developed, with detection limits of <10^2^ copies/μL sample [3]. Using conventional PCR, PCV3 was detected in the Malaysian commercial pig population from 2016 to 2019, with molecular prevalence rates of 41.67% and 17.02% in farms and domestic pig populations, respectively. The molecular detection rate was highest in the inguinal lymph nodes and lungs, at 90% and 73.33%, respectively [11]. At present, the development of PCV3 antibodies and their subsequent application for direct antigen detection is limited to studies involving immunohistochemistry (IHC) and immunofluorescence assay (IFA) [3]. Under these circumstances, *in situ* hybridization (ISH) assay might be a valuable adjunct to the current molecular antigen detection methods for investigating tissue tropism and pathogenesis of PCV3. In general, ISH involves fixation and pre-treatment of tissue samples to preserve and present the target nucleic acid optimally. Then, the labeled probes are hybridized using complementary base pairing to the target, which is detected using microscopic visualization of the hybrid [12]. Successful localization of PCV3 antigen within the tissue environment is crucial for elucidating its pathogenesis. Although ISH has been used in several PCV3 studies, it has not yet been thoroughly described. Furthermore, most PCV3 ISH studies employed the RNAScope assay, which might not be economically feasible for small-scale research or diagnostics laboratories [2, 13, 14].

This study aimed to describe a chromogenic ISH technique using digoxigenin (DIG)-labeled cloned PCV3 open reading frames 1 (ORF1) fragment DNA to detect the localization of PCV3 antigen in formalin-fixed, paraffin-embedded (FFPE) lung, and lymphoid tissue specimens.

## Materials and Methods

### Ethical approval

The study was approved by the Institutional Animal Care and Use Committee (IACUC) under AUP Code UPM/IACUC/AUP-R030/2019 and was conducted adhering to the guidelines as stated in the Code of Practice for Care and Use of Animals for Scientific Purposes as stipulated by Universiti Putra Malaysia.

### Study period and location

The study was conducted from June to November 2020 in College of Veterinary Medicine, National Pingtung University of Science and Technology, and in Faculty of Veterinary Medicine, Universiti Putra Malaysia.

### Samples

We selected a sample subset with ten corresponding sets of lung and lymphoid tissues from a previous Malaysian PCV3 study based on the PCV3 PCR results on fresh tissues and the availability of FFPE tissues [11]. All the PCV3-positive and -negative samples were also positive for PCV2 and porcine reproductive and respiratory syndrome (PRRS) and negative for PCV4. Porcine circovirus 2, PCV3, and PCV4 antigens were detected using PCR, as described previously by Tan *et al*. [8, 11, 15]. The PCR was done to detect the PRRS viral antigen using the RealPCR PRRSV Type 1 and Type 2 Multiplex RNA Mix (IDEXX, US) according to the manufacturer’s instructions. The fresh tissues were kept at −80°C (Haier Thermo Scientific TDE60040LD Ultra-Low Temperature Freezer, China) in the Clinical Research Laboratory, Faculty of Veterinary Medicine, Universiti Putra, Malaysia. A part of each sample was preserved as FFPE tissues. The tissue samples were cut into small pieces (1 cm × 1 cm), fixed in 10% (w/v) neutral buffered formalin for 48 h, and paraffin-embedded according to standard histopathological procedures. Then, 4 µm-thick sections were cut using a microtome (Leica HI1210, Germany) and floated on a 45°C water bath. The resulting tissue section ribbons were mounted on poly-L-lysine coated glass slides for ISH (PorLab Scientific, China).

### In situ hybridization

#### Preparation of DIG-labeled probe

We subjected two PCV3-positive samples from the previous Malaysian PCV3 study to conventional PCR to amplify a 69 bp region of the PCV3 ORF1 spanning from nucleotide (nt) positions 282 – 350. The PCR was performed using the same reagents and reaction volumes described in the PCV3 cap gene detection methodology [11]. Table-1 provides the details of the primer pair and cycling conditions.

**Table-1:** Primers and PCR cycling conditions used in this study for amplification of PCV3 ORF1 fragment.

Primer pair	Nucleotide Sequence (5’– 3’)	Product length (bp)	PCR cycling condition (temperature/time)					

			Initial denaturation	Number of cycle	Denaturation	Annealing	Extension	Final extension
PCV3-69F	CGAGTGGGAATCTATTGTGGA	69	95°C/3 min	40	98°C/20 s	50°C/30 s	72°C/50 s	72°C/10 min
PCV3-69R	CCAACCTCTTTGCCGATAAT							

PCR=Polymerase chain reaction, PCV3=Porcine circovirus 3, ORF1=Open reading frame 1

Agarose electrophoresis of the PCR products showed a positive band between 50 and 100 bp region as marked by GelPilot 50 bp Plus Ladder (Qiagen^®^, Germany). These bands were purified using the MEGAquick-spin™ Total Fragment DNA Purification Kit (Intron^®^, Korea) and sequenced (Macrogen Inc., South Korea). The MEGA v7.0.26 software program was used for sequence assembly and multiple sequence alignment [16]. The resulting sequences were analyzed using NCBI Nucleotide BLAST^®^ [17] to confirm PCV3 by comparing their similarity with reference PCV3 sequences from GenBank.

Using the purified PCR product as the insert DNA, PCR cloning was performed using the StrataClone PCR Cloning Kit according to the manufacturer’s instructions with some modifications (Agilent^®^, US). First, the ligation reaction mixture was prepared by gently mixing 3 μL of cloning buffer, 2 μL of the aforementioned purified PCR product (50 ng), and 1 μL of pSC-A-amp/kan cloning vector. Individual tubes of StrataClone SoloPack competent cells were thawed on ice immediately after removing them from the freezer. After 20 min of incubation at 24–26°C, 1 μL of the ligation mixture was gently mixed by swirling it into the thawed competent cells. After incubating for 20 min on ice, the transformation mixture was heat-pulsed at 42°C for 45 s and promptly kept on ice for 2 min. Next, 200 μL of super optimal broth with catabolite repression (SOC) medium, pre-warmed at 42°C, was added to the tubes containing the transformation mixture (Sigma-Aldrich, US), which were placed horizontally in an incubator shaker for 1 h at 37°C with 1 × *g* agitation. The media was prepared using 20 g of Miller’s LB broth powder (Huankai Microbial, China) and 20 g of nutrient agar powder (HiMedia, US) in 1L of deionized water. After the media was autoclaved and cooled down to 55°C, 10 mL of 10 mg/mL filter-sterilized ampicillin (ampicillin: Bio Basic, Canada; 25 mm/0.22 µm syringe filter: CNW, China) was added into the media. For each 100 mm Petri dish, 25 mL of agar was poured and cooled at 24–26°C for 30 min.

For the blue-white screening, 2% X-gal was prepared by mixing 0.2 g X-gal in 10 mL IPTG (Sangon Biotech, China) and adding it onto the LB-ampicillin plates at 40 μL per plate. For each transformation mixture, 5 μL and 100 μL volumes were plated separately. The 5 μL mixture was plated by pipetting it into 50 μL of SOC media to facilitate spreading. The X-gal and transformation mixture were uniformly plated using 15 autoclaved glass plating beads for each Petri dish (Zymo Research, US). The plates were gently shaken in different directions to evenly roll the beads. After the agar surface dried, the beads were removed, and the plates were incubated for 24 h at 37°C. After the transformation, the white colonies were inoculated into LB-ampicillin broth overnight in a shaker incubator at 37ºC and 3 × *g*. After the incubation, 10 mL of culture from each incubated cloning reaction was collected for plasmid isolation. Plasmid DNA isolation was performed using ISOLATE II Plasmid Mini Kit, according to low-copy plasmid isolation protocol (Meridian Life Science, US).

Next, 25 pg of the purified plasmid DNA was used as the template DNA for DIG-dUTP (DIG-dUTP, Sigma-Aldrich) labeling. The reaction mix was prepared using the PCR DIG Probe Synthesis Kit (Sigma-Aldrich) according to the manufacturer’s instructions using the cycling conditions shown in Table-1. The end reaction mixture was verified for successful DIG-labeled probe synthesis by agarose gel electrophoresis with 5 μL of PCR product stained with GelPilot DNA loading dye as molecular weight marker (Qiagen).

#### *In situ* hybridization

*In situ* hybridization was performed using the BioSpot ISH Kit for the DIG probe with the modified protocol (BioTnA, Taiwan). The slides were heated on a heating plate at 60°C for 20 min until the wax dissolved. For deparaffinization of the tissue sections, the slides were rinsed thrice with absolute xylene for 5 min each and rehydrated for 5 min each through a 100%, 90%, 70%, 50%, and 30% ethanol gradient. After washing with distilled water, hydrogen peroxide block was dropped liberally on the slides, and coverslips were placed on the tissue sections. After incubation for 20 min at 25°C, the slides were rinsed with tris-buffered saline solution, which was used for all subsequent rinsing steps. Next, the slides were immersed in heat pre-treatment solution pre-warmed to 100°C and maintained in an isothermal water bath for 30 min. The slides were cooled for 5 min and then rinsed. Next, pepsin solution pre-warmed to room temperature was dropped on the slides, covered with coverslips, incubated in a moist humidity chamber at 37°C for 10 min, and rinsed. After pre-treatment, the ISH probe was prepared as a 1/150 dilution: 1 µL probe to 150 µL of hybridization buffer (Roche, Switzerland). After heating the diluted probe in a 100°C water bath for 15 min, 100 µL of the heated probe was added to each slide, and coverslips were placed over the tissue sections. The slides were incubated in a moist humidity chamber at 55°C for 16 h, immersed in saline sodium citrate solution pre-warmed to 55°C, and maintained in an isothermal water bath for 5 min. After rinsing, blocking solution was added to the slides, covered with coverslips, incubated in a moist humidity chamber at 37°C for 30 min, and rinsed. The step was repeated using a mouse anti-DIG antibody for a longer incubation period of 60 min. Horseradish peroxidase was applied on the slides, incubated at 25°C for 30 min, and rinsed. The slides were stained with DAB for 20 s and counterstained with nuclear blue stain for 15 min. Finally, they were dehydrated for 5 min each through 30%, 50%, 70%, 90%, and 100% ethanol and evaluated using light microscopy evaluation using an imaging microscope (Motic Image plus 2.0, China).

## Results and Discussion

**Figure-1:**
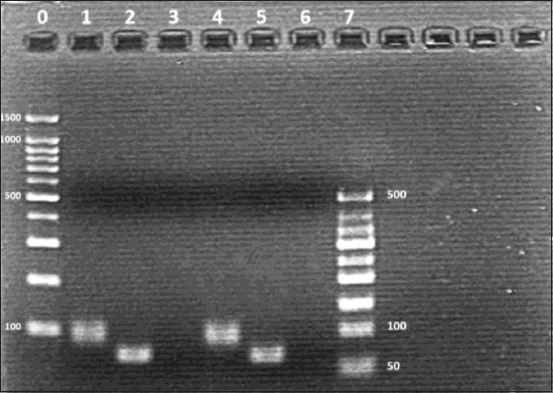
Verification of in situ hybridization (ISH) digoxigenin (DIG) probe development by agarose electrophoresis. DIG-labeled probes were successfully developed using porcine circovirus 3 (PCV3) positive samples L43 and L53 as templates. Unlabeled probes were expected to be 69 bp, while corresponding labeled probes were expected to appear larger. The generated probes were used in subsequent ISH staining protocol. Lane 0: DNA ladder (Qiagen^®^ GelPilot 50 bp ladder). Lane 1: Positive band for unlabeled experimental PCV3 probe from sample L43. Lane 2: Positive band for DIG-labeled experimental PCV3 probe from sample L43. Lane 3: Negative control. Lane 4: Positive band for unlabeled experimental PCV3 probe from sample L53. Lane 5: Positive band for DIG-labeled experimental PCV3 probe for sample L53. Lane 6: Negative control. Lane 7: DNA ladder (Qiagen^®^ GelPilot 100 bp plus ladder).

Nucleotide sequence analysis of the PCR products showed the targeted ORF1 region from nt position 282 to 350 for positive samples L43 and L53. Agarose electrophoresis results indicated a positive band between the 50 and 100 bp region for the unlabeled control probes, while the corresponding DIG-labeled probes were closer to 100 bp (Figure-1). The DIG-labeled probes were expected to migrate slower during electrophoresis as the DIG molecules increased the overall molecular weight. Hence, the development of a PCV3-specific ISH DIG probe targeting the 69 bp region of PCV3 ORF1 was confirmed.

**Figure-2:**
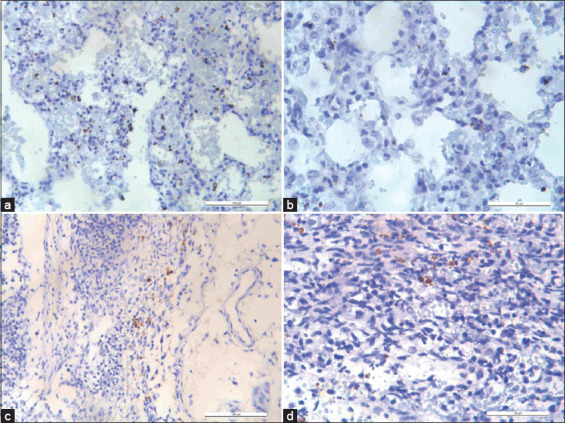
(a) Positive *in situ* hybridization (ISH) results from lung tissues. Multifocal ISH positive signals were observed as brown grains of chromogenic staining within cytoplasm of pneumocytes. 200× magnification with a scale bar equivalent to 100 µm. (b) Positive ISH results from lung tissues. Multifocal ISH positive signals were observed as brown grains of chromogenic staining within cytoplasm of pneumocytes. 400× magnification with scale bar equivalent to 50 µm. (c) Positive ISH results from inguinal lymph node tissue. Multifocal ISH positive signals were observed as brown grains of chromogenic staining within cytoplasm of lymphocytes. 200× magnification with scale bar equivalent to 100 µm. (d) Positive ISH results from mesenteric lymph node tissues. Multifocal ISH positive signals were observed as brown grains of chromogenic staining within cytoplasm of lymphocytes. 400× magnification with scale bar equivalent to 50 µm.

**Figure-3:**
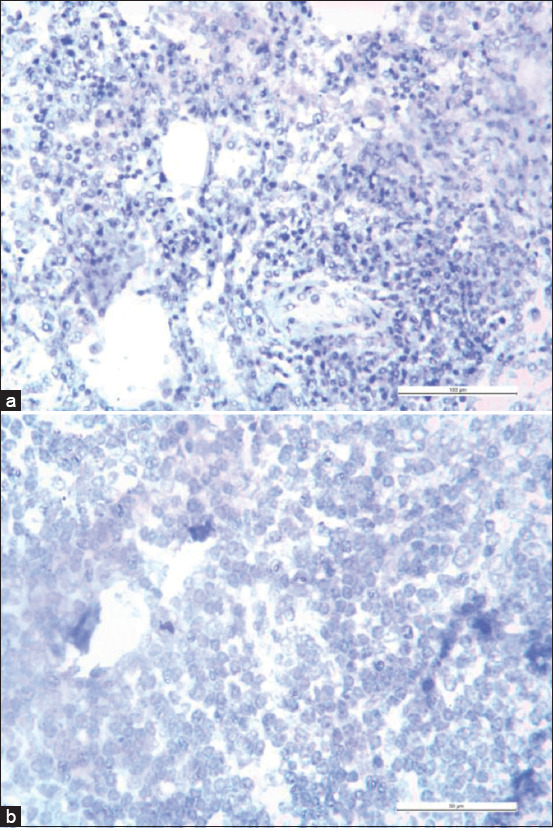
(a) Negative *in situ* hybridization (ISH) results from lung tissues. *In situ* hybridization positive signals were not observed. 200× magnification with scale bar equivalent to 100 µm. (b) Negative ISH results from inguinal lymph node tissues. *In situ* hybridization positive signals were not observed. 400× magnification with scale bar equivalent to 50 µm.

Light microscopic observation of PCV3 antigens that were stained brown indicates positive ISH results. Figures-2a-d showed positive ISH staining results in the lung, inguinal lymph node, and mesenteric lymph node tissue sections under magnification, while Figures-3a and b showed negative ISH staining results.

As the previous Malaysian PCV3 study [11] reported that PCV3 was mainly detected in the inguinal lymph nodes and lung tissues, we targeted these tissues in this study. We also collected tissue samples from these organs for the cases with PCV3-suspected disease, together with heart tissues [18]. All the positive hybridization signals observed during PCV3 ISH were intracytoplasmic with a predominantly multifocal distribution pattern, consistent with the previous ISH studies [14, 19]. Apart from lung and lymph node tissues, positive ISH signals were also reported in lesions of various organs, including myocarditis, arteritis, perivasculitis, nephritis, chorioamnionitis, encephalitis, dermatitis and pneumonia from fetuses, weaners, growers, and sows [2, 13, 14, 18–21]. Table-2 shows the results of PCV3 molecular detection status using PCR and ISH, which were in good agreement.

**Table-2:** Tabulation of PCV3 ISH results compared to PCV3 PCR results. Samples were segregated based on PCV3 PCR detection status to compare agreement between PCV3 PCR and ISH detection results.

PCV3 PCR positive samples (n = 11)			PCV3 PCR negative samples (n = 19)		
	
PCV3 PCR results	Sample ID	PCV3 ISH Results	PCV3 PCR results	Sample ID	PCV3 ISH results
PCV3-positive	43 - ILN	Positive	PCV3-negative	43 - Lung	Negative
	43 - MLN	Negative		208 - Lung	Negative
	51 - Lung	Positive		208 - ILN	Negative
	51 - ILN	Positive		208 - MLN	Negative
	51 - MLN	Positive		209 - Lung	Negative
	53 - Lung	Positive		209 - ILN	Negative
	53 - ILN	Positive		209 - MLN	Negative
	53 - MLN	Positive		210 - Lung	Negative
	199 - Lung	Positive		210 - ILN	Negative
	199 - ILN	Positive		210 - MLN	Negative
	199 - MLN	Positive		220 - Lung	Negative
				220 - ILN	Negative
				220 - MLN	Negative
				221 - Lung	Negative
				221 - ILN	Negative
				221 - MLN	Negative
				222 - Lung	Negative
				222 - ILN	Negative
				222 - MLN	Negative

ILN=Inguinal lymph node, MLN=Mesenteric lymph node, PCR=Polymerase chain reaction, PCV3=Porcine circovirus 3, ISH=*In situ* hybridization

Only one out of the 11 samples positive for PCV3 by PCR method was ISH-negative. This was probably because the mesenteric lymph node tissues collected during post-mortem were too scarce, causing imperfect tissue fixation onto the ISH slide and insufficient binding of the DIG probe to the tissues. The PCR and ISH results for all 19 PCV3-negative samples were in agreement. Further, no false positivity of PCV2 and PRRS was observed. No positive ISH signals were observed in the PCV3-negative samples, which were positive for PRRS and PCV2.

At present, PCV3 detection is mostly done using molecular techniques, such as conventional PCR and qPCR, and its characterization by Sanger sequencing or next-generation sequencing (NGS) [22]. Five years into its discovery, commercial PCV3 antibodies are still not widely available, possibly due to limited virus isolation [3]. This limited availability of PCV3-specific antibodies restricts the development of IHC and IFA methods. Compared to IHC and IFA that require antibodies, ISH can be developed more rapidly using only the sequence information from tissue samples positive for PCV3. Furthermore, ISH can be applied in conjunction with PCR or qPCR, NGS, and histopathology to investigate the association among pathogens, lesions, and clinical outcomes [23]. Disease investigation or epidemiological study can start with PCV3 detection and/or quantification by PCR or qPCR, followed by ISH to visualize the localization of PCV3 DNA within tissues or cells.

Successful ISH depends on sufficient preservation of the sample tissues’ morphologic details, the ISH probe’s specificity, proper probe hybridization, and labeling for accurate antigen detection [24]. All the samples in this study were fixed in 10% neutral buffered formalin for 48 h immediately on collection to maximize the detection potential of nucleic acids within FFPE tissues [25]. The ISH results are also less susceptible to structural alterations in tissue, commonly seen with formalin fixation [26, 27]. Although ISH with RNA probes is more sensitive than DNA probes, given the single-stranded nature of the PCV3 DNA, DNA probes are considered equally sensitive and reliable as RNA probes for diagnostic purposes [26, 28]. The ISH probes in this study were designed based on the ORF1 gene of PCV3, considering that it is the most conserved region of the circovirus genome [29]. Further, the probe length was kept to a minimum of 69 bp to ensure the specificity of the probe. The ISH procedure, from cloning to prepare the DIG-labeled probes to probe hybridization and labeling, was optimized to achieve accurate PCV3 antigen detection. Further, compared to the antibodies used for IHC, the antibodies for the DIG-labeled probes do not show any cross-reactivity with animal tissues [25]. Thus, by combining DIG-labeled DNA probes and ISH in this study, we minimized the possibility of errors caused by antigenic cross-reactivity and/or the alteration of binding sites caused by formalin fixation [30]. Moreover, most of the published PCV3 ISH studies used the RNAScope assay, which is more expensive [2, 13, 14]. In contrast, our DIG-ISH protocol is a more economically feasible and practical option for smaller-scale research or diagnostics laboratories.

## Conclusion

This study reports a chromogenic ISH technique using DIG-labeled cloned PCV3 ORF1 fragment DNA probe that enabled the detection and localization of PCV3 antigen in FFPE lung and lymphoid tissue specimens. Here, PCR and ISH results for detecting PCV3 were in good agreement. Only one lymphoid tissue sample was ISH-negative, presumably due to lower tissue availability. *In situ* hybridization can complement PCR in detection studies for the localization of antigens in infected tissue sections. The diagnostic application of ISH could remain relevant in investigating PCV3 replication and pathogenesis, especially with the limited development of PCV3 antibodies required by other immunostaining methods.

## Authors’ Contributions

CYT and KCL: Sample and data collection. CYT, KCL, MTC, CNL and PTO: Study design and drafted the manuscript CYT and KCL: Laboratory work, data analysis, and drafted the manuscript. All authors have read, reviewed, and approved the final manuscript.
